# Evolutionary origins of taste buds: phylogenetic analysis of purinergic neurotransmission in epithelial chemosensors

**DOI:** 10.1098/rsob.130015

**Published:** 2013-03

**Authors:** Masato Kirino, Jason Parnes, Anne Hansen, Sadao Kiyohara, Thomas E. Finger

**Affiliations:** 1 Department of Chemistry and BioScience, Graduate School of Science and Engineering, Kagoshima University, Kagoshima, Japan; 2 Department of Cell and Developmental Biology, Rocky Mountain Taste and Smell Center, University of Colorado School of Medicine, MS 8108, Room L18-11118, RC-1, 12801 E. 17th Avenue, Aurora, CO 80045, USA

**Keywords:** taste, evolution, purinergic signalling, ENTPDase, Schreiner organ, solitary chemosensory cell

## Abstract

Taste buds are gustatory endorgans which use an uncommon purinergic signalling system to transmit information to afferent gustatory nerve fibres. In mammals, ATP is a crucial neurotransmitter released by the taste cells to activate the afferent nerve fibres. Taste buds in mammals display a characteristic, highly specific ecto-ATPase (NTPDase2) activity, suggesting a role in inactivation of the neurotransmitter. The purpose of this study was to test whether the presence of markers of purinergic signalling characterize taste buds in anamniote vertebrates and to test whether similar purinergic systems are employed by other exteroceptive chemosensory systems. The species examined include several teleosts, elasmobranchs, lampreys and hagfish, the last of which lacks vertebrate-type taste buds. For comparison, Schreiner organs of hagfish and solitary chemosensory cells (SCCs) of teleosts, both of which are epidermal chemosensory end organs, were also examined because they might be evolutionarily related to taste buds. Ecto-ATPase activity was evident in elongate cells in all fish taste buds, including teleosts, elasmobranchs and lampreys. Neither SCCs nor Schreiner organs show specific ecto-ATPase activity, suggesting that purinergic signalling is not crucial in those systems as it is for taste buds. These findings suggest that the taste system did not originate from SCCs but arose independently in early vertebrates.

## 2. Introduction

Taste buds are the gustatory end organs in vertebrates ranging from lamprey to mammals [1]. These end organs respond to a variety of sapid chemicals, and transmit signals to afferent nerves fibres arising from three cranial ganglia: facial, glossopharyngeal and vagus. While other nerves, e.g. trigeminal, may heavily invest the epithelium surrounding the taste buds (the so-called perigemmal innervation), the trigeminal fibres do not enter the taste bud itself. Taste buds can be recognized by three key features. (i) Taste buds are an aggregate of elongate taste cells of multiple morphological and functional types. (ii) Taste cells extend from the basal lamina to an apical pore or other opening in the epithelium. (iii) Finally, taste buds are innervated by sensory fibres of the cranial nerves containing cells derived from epibranchial placodes: i.e. the facial, glossopharyngeal or vagus nerves [2].

Throughout the vertebrate lineage, numerous epithelial chemoreceptors can be identified, but only taste buds meet the three criteria listed above. For example, most fishes possess solitary chemosensory cells (SCCs) scattered across the body surface [3]. SCCs share some features with taste buds, i.e. they are elongate, span the height of the epithelium, form synapses with afferent nerves and even may express common receptors [4,5]; but SCCs are not merely dispersed taste cells. The SCCs mostly appear singly, not in clusters, and, unlike taste buds, can be innervated by non-gustatory (e.g. spinal or trigeminal) nerves [3,6,7]. Previously, some have speculated that SCCs may even be phylogenetic forerunners of taste buds [8]. Hagfish possess another distinct chemosensory endorgan, the Schreiner organ, which shares many features with taste buds, but again is not identical to taste buds [4]. Schreiner organs are an assembly of several cell types and form functional contacts with nerve fibres entering from their basal aspect. But Schreiner organs do not extend to the basal lamina and can be innervated by non-gustatory nerves, e.g. the trigeminal nerve [4]. Thus, Schreiner organs are not considered to be taste buds, but may be functionally or phylogenetically related to taste buds.

An unusual feature of taste buds, at least in mammals, is their dependence on purinergic neurotransmission to convey the signal from taste buds to the nervous system [9]. Taste cells release ATP upon stimulation by tastants [9–12] and the gustatory nerve fibres express two ionotropic purinergic receptors, P2X2 and P2X3, which are required for activation [9]. Many taste cells express a highly specific ecto-ATPase (NTPDase2) [13], necessary for inactivation of the purinergic neurotransmitter. This ectonucleotidase isoform is highly selective for ATP over ADP [14] and so can be identified by classical histochemical methods employing these two different substrates. As nucleotidase activity has been reported in teleost taste buds [15], it is likely that taste buds in teleosts also employ purinergic signalling. The present study was undertaken to test whether taste buds in all vertebrates use purinergic neurotransmission, and whether this unusual mechanism is also associated with other epithelial chemoreceptor systems.

Ultrastructural and immunochemical studies show that taste buds consist of a heterogeneous population of cells. Mammalian taste buds cells are classified into three principal types of mature, elongate cells, i.e. type I, II and III, along with proliferative basal cells lying just outside the taste bud proper [8,16–18]. The elongate cells extend an apical process into an opening in the epithelium, and a basal process, which may reach the basal lamina of the epithelium. Although the cell types were originally described based purely on ultrastructural criteria [19], each of the cell types appears to have a unique function. In mammals, the type II cells express metabotropic taste receptor proteins and elements of the G-protein transduction cascade required for sweet, bitter and umami tastes [20–22]. Similarly, type III cells express the channels used in sour and possibly salt transduction [23,24]. The type III and type II cells apparently transmit this information to the gustatory nerves through the agency of ATP acting on P2X-type ATP receptors situated on the nerve fibres [9]. Type I cells are considered to be supporting cells like glia because type I cells often envelop other elongated cells and express proteins such as GLAST, which are expressed by glia in the central nervous system. More importantly, the type I cells also express the ecto-ATPase (NTPDase2) highly specific for ATP [13,14]. Presumably, this ecto-ATPase is crucial in inactivating the ATP signal release by the taste cells. Thus, the presence of this specific ATPase can serve as a marker for the utilization of ATP as a neurotransmitter in the taste bud system.

In fish, the cellular organization of taste buds is less well understood, but consists of at least three types of cells [15,25,26]. According to their morphological features, they are termed tubular or light cells, filamentous or dark cells and basal cells. Multiple classes of light cells have been reported [27]. Both light and dark cells are elongate cells and are generally believed to be receptor cells and supporting cells, respectively. One prominent type of basal cell in fish taste buds are Merkel-like in terms of morphology and neurotransmitter content [8,15]. In addition, proliferative marginal cells lie along the basolateral margin of the taste bud similar to basal cells of mammalian systems [27]. Light cells, dark cells and Merkel-like basal cells all have synaptic connection to afferent fibres [15]. Synaptic neurotransmitters in taste buds of fish are unknown although the Merkel-like basal cells accumulate and presumably release serotonin, similar to type III cells of mammalian taste buds [28] and Merkel-like basal cells in amphibia [29].

The purpose of this study was to determine when during the evolutionary history of taste buds and epithelial chemoreceptor cells a highly specific ecto-ATPase appeared. Is the presence of ecto-ATPase coincident with the appearance of vertebrate taste buds, or is ecto-ATPase present in or around other epithelial chemosensory endorgans suggestive of a more ancient origin for ATP neurotransmission by chemosensory systems? The present results show that ecto-ATPase is present in taste buds of fish including lamprey but not at all in Schreiner organs in hagfish, nor is it associated with SCCs of any vertebrate. These findings suggest that utilization of ATP as a transmitter co-evolved with the taste system in early vertebrates.

## 3. Material and methods

### 3.1. Animals

In this study, we examined six species of Teleostei (channel catfish, *Ictalurus punctatus*; sea catfish, *Plotosus japonicus* (*n* = 10); Japanese sea robin, *Chelidonichthys spinosus* (*n* = 6); common carp, *Cyprinus carpio* (*n* = 3); goldfish, *Carassius auratus* (*n* = 4); and zebrafish line P2X3.2 : gfp [30] (*n* = 2)), as well as one elasmobranch species (cat shark, *Scyliorhinus torazame* (*n* = 2))*,* two species of lamprey, *Lethenteron japonicum* (*n* = 4) and *Petromyzon marinus* (*n* = 10), and one species of hagfish, *Eptaretus burgeri* (*n* = 10). Apart from the transgenic zebrafish, all species were obtained commercially or caught with fisheries nets except for *P. marinus*: tissue was kindly supplied by Sorensen (University of Minnesota) and amnocetes by Nicholas Johnson, Hammond Bay Biological Station (Millersburg, MI, USA). The P2X3.2 : gfp line of zebrafish was generously supplied by Mark Voigt (St Louis University) and maintained at the University of Colorado Anschutz campus aquatics facility. The experiments for zebrafish, channel catfish, goldfish and lamprey were performed at the University of Colorado, School of Medicine and for others at Kagoshima University. All experiments were carried out with the approval of the local animal care and use committees (University of Colorado IACUC or guidelines of Kagoshima University).

### 3.2. Enzyme histochemistry for light microscopy

Ecto-ATPase activity was examined by lead precipitation as described previously [31,32]. Specific ATPase activity was determined by comparison of tissues reacted with 1 mM ATP substrate compared with those reacted with 1 mM ADP substrate. Purinergic signalling and ecto-ATPase enzymes are phyletically ancient, present even in singe-celled organisms [33]. Among vertebrates, the different ectonucleotidase isoforms are characterized in part by their substrate specificity. The ecto-ATPase associated with taste buds in mammals, NTPDase2, is highly specific for ATP over ADP or AMP and this property distinguishes this isoform from other, related ectonucleotidases [14]. Hence, histochemical results comparing hydrolysis of ATP with ADP is strongly indicative of NTPDase isoform. In this paper, we refer to enzyme activity particular to the ATP substrate but absent with the ADP substrate as ecto-ATPase.

Each species of fish was deeply anaesthetized with dilute tricane methansulfonate (MS222), and either fixed by transcardial perfusion or with immediate removal of chemosensory tissues from the specimen. Tissues were immersed for up to 1 h in 2 per cent paraformaldehyde, 0.2 per cent glutaraldehyde, in 0.1 M Tris–maleate buffer (pH 7.4) with 2 mM CaCl_2_ and then placed overnight in the same buffer at 4°C with 20 per cent sucrose for cryoprotection. The tissues were cut on a freezing microtome or cryostat at 12–40 μm. Free-floating sections or slides were rinsed three times for 10 min each with 0.07 M Tris–maleate buffer (pH 7.4).

In the beginning of the experiments, histochemical tests of ATPase were performed in the barbels of *Plotosus* and *Carrassius*. The free-floating sections of barbels, lips or palatal organ were incubated first with the following medium for 30 min at room temperature: 2 mM Pb(NO_3_)_2_, 5 mM KCl, 2 mM CaCl_2_ and 1 mM of substrate, either ATP or ADP. The incubation was followed by three 10-min washes in 0.07 M Tris–maleate buffer. The lead precipitate was visualized by treating the sections for 1 min with 1 per cent ammonium sulfide. After several rinses in distilled water, the sections were collected on slides, then coverslipped with fluoromount (Fisher Biotec). Some sections were counterstained with Giemsa dye. The sections were viewed under a Nikon or Olympus light microscope. After observation of significant activities in the presence of ATP, the incubation medium was modified; three kinds of inhibitors were added to the above mentioned medium: 1 mM levamisole (inhibits alkaline phosphatases), 1 mM ouabain (inhibits Na^+^,K^+^-ATPase) or 50 μM α,β-methylene ADP (inhibits 5′-nucleotidase). Incubation medium without CaCl_2_ was also tested.

**Figure 1.**
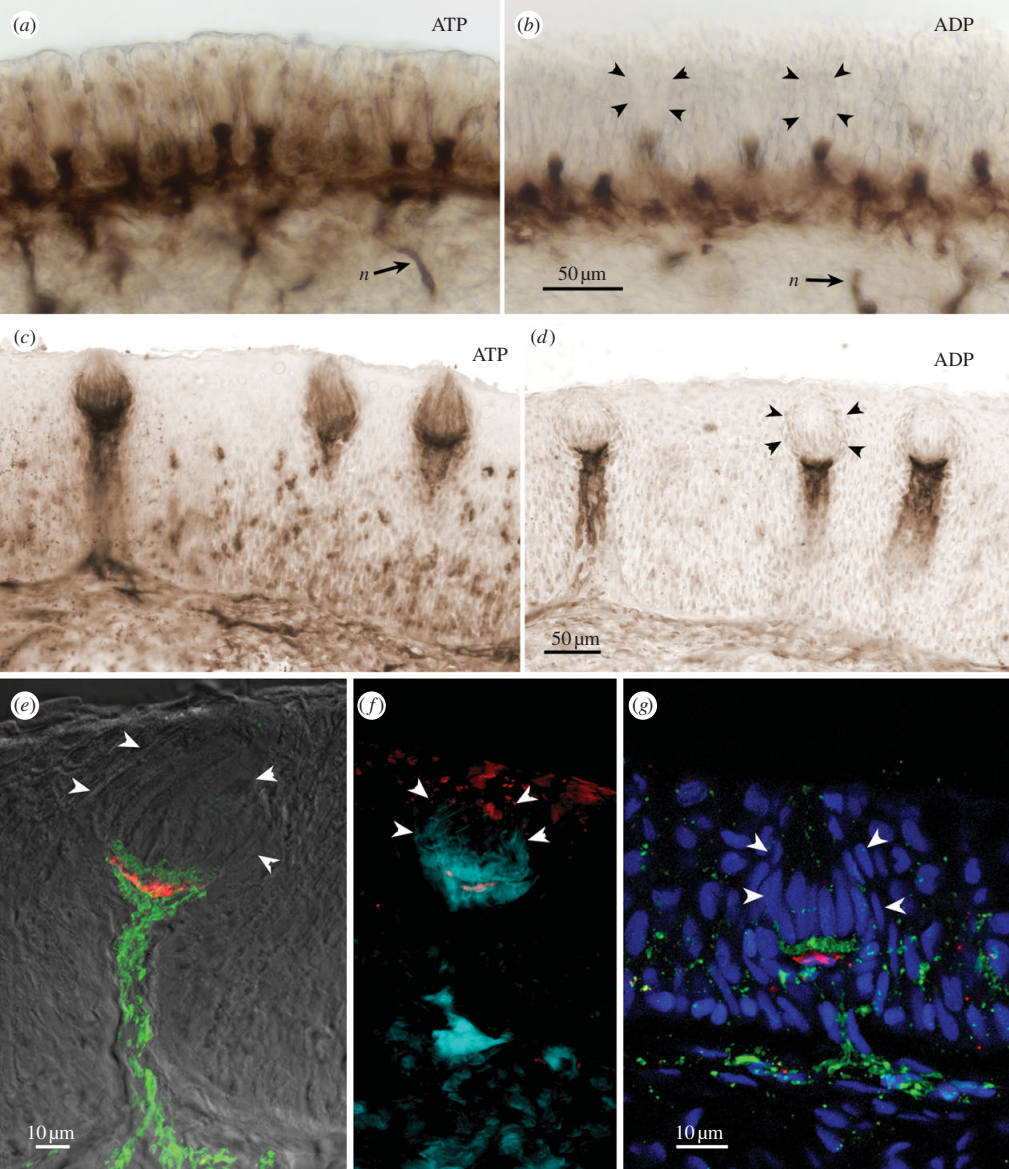
(*a–f*) Ecto-ATPase and non-specific nucleotidase (ADP) staining in taste buds from goldfish, *C. auratus*. (*a,b*) Sections through the palatal organ showing (*a*) specific ecto-ATPase activity, and (*b*) non-specific nucleotidase activity. (*c,d*) Sections through the lip showing (*c*) specific ecto-ATPase activity, and (*d*) non-specific nucleotidase activity. In (*a–d*), Ecto-ATPase staining is evident in the elongate cells of the taste bud as well as in the basal portion of the taste bud proper. Non-specific staining with ADP as substrate (*b,d*) shows reaction product surrounding the vertical nerve bundles and extending to a pedestal below the taste bud. (*e,f*) Higher magnification views of single taste buds showing serotonergic immunoreactivity (red) of the (*e*) Merkel-like basal cells in lip and (*f*) palatal organ. The ecto-ATPase activity is shown in pseudocolour: green in (*e*) and aqua in (*f*). The pseudocolour image is produced by inverting a brightfield image and placing into a colour channel of the composite image from tissue first reacted for ecto-ATPase and then immunoreacted for serotonin. Note that the ecto-ATPase staining appears both above and below the Merkel-like basal cell. (*g*) Longitudinal section through the taste bud from the lip of a P2X3a-GFP zebrafish showing that nerve fibres expressing purinergic receptors (green) form a plexus mostly above the Merkel-like basal cell immunoreacted for serotonin (red). In all panels, arrowheads indicate the edge of the taste bud.

Optimal conditions for the specific detection of ecto-ATPase involved fixation times of 30–60 min followed by prolonged washes in buffer. These optimal reaction conditions were used in processing tissues from the diverse species. Specific ecto-ATPase activity described below is defined as reaction product observed with ATP in the presence of inhibitors of other phosphatases and nucleotidase, but not the ADP substrate in the same reaction medium (figure 1*a–d*).

### 3.3. Enzyme histochemistry for electron microscopy

On one specimen each of goldfish and catfish (*I. punctatus*), we carried out the ATPase reaction for electron microscopy slightly modified from the protocol of Barry [31]. As the lead precipitate is electron dense, little modification is necessary. Fixation was as for light microscopy, but after fixation the tissue was cryoprotected in an ascending series of glycerin/sucrose in Tris–maleate buffer ending with 15 per cent glycerin, 20 per cent sucrose [34]. Free-floating 40 µm sections were cut on a cryostat into 70 mM Tris buffer and were then reacted as for light microscopy using either ATP or ADP as substrates. Following the reaction, sections were not placed into ammonium sulfide but rather were postfixed in 4 per cent EM grade glutaraldehyde in cacodylate buffer. After overnight postfixation, the tissue was rinsed in cacodylate buffer and placed into 1 per cent osmium tetroxide for 30 min. After rinsing in cacodylate buffer, the specimens were dehydrated in a graded series of ethanol and propylene oxide and embedded in Epon-Araldite (Electron Microscopy Sciences, Hatfield, PA, USA). Ultrathin sections (silver to gold) were stained with uranyl acetate and lead citrate and examined with a FEI Tecnai G2 electron microscope (Philips, Eindhoven, The Netherlands).

### 3.4. Immunohistochemistry

In order to delineate the different types of cells and innervation of taste buds, we carried out a series of immunohistochemical experiments in tissues from some teleosts and the lamprey. In all cases, omission of the primary antiserum yielded no specific reactivity reported below.

#### 3.4.1. Single-label staining after ATPase reaction

Following distilled water rinses, the sections were washed for 10 min in 0.1 M PB (phosphate buffer, pH 7.2–7.4) and then 2 × 10 min changes of 0.1 M PBS (phosphate-buffered saline, pH 7.2–7.4). The tissue was then incubated for 1 h in blocking solution (3% normal donkey serum, 1% bovine serum albumin, 0.3% Triton in PBS) before an overnight incubation in rabbit anti-serotonin (1 : 5000, lot: 924005, Immunostar, Hudson, WI, USA) or (for zebrafish) chicken anti-GFP (1 : 2000, lot: 0609FP10, Avēs labs, Tigard, OR, USA) diluted in blocking solution at 40°C. After 3 × 10 min washes in 0.1 M PBS, the sections were incubated in secondary antibodies for 2 h at room temperature: DyLight 550 anti-rabbit (1 : 500, lot: GR32373–2, Abcam) or Alexa 488 anti-chicken (1 : 500, lot: 102758, Life Technologies, Gand Island, NY, USA). A far red draq5 counterstain was applied during the secondary antibody application (1 : 1000, lot: 402DR50050, Abcam). Slides were then coverslipped in Fluoromount-G.

#### 3.4.2. Double-label staining

Alternate sets from the ATPase tissue were used for immunohistochemistry. Slides were washed 3 × 10 min in 0.1 M PBS and incubated for 1 h in blocking solution. Overnight incubation of the primary antibodies, mouse anti-acetylated tubulin (1 : 5000, lot: 118K4821, Sigma, St Louis, MO, USA), rabbit anti-serotonin (1 : 5000, lot: 924005, Immunostar) or chicken anti-GFP (1 : 2000, lot: 0609FP10, Avēs labs) was carried out at 4°C. Three 10 min washes in 0.1 M PBS preceded secondary incubation for 2 h at room temperature: DyLight 550 anti-rabbit (1 : 500, lot: GR32373–2, Abcam) or Alexa 488 anti-chicken (1 : 500, lot: 102758, Life Technologies) or Alexa 488 anti-mouse (1 : 500, lot 811493, Life Technologies) was diluted in the blocking solution. A far red draq5 counterstain was applied during the secondary antibody application (1 : 1000, lot: 402DR50050, Abcam). Slides were then coverslipped in Fluoromount-G after three more rinses in 0.1 M PBS.

## 4. Results

### 4.1. Ecto-ATPase in teleosts

Taste buds in teleosts reacted strongly for ecto-ATPase activity, but the labelling was slightly different for taste buds innervated by the facial nerve (lips and barbels) compared with taste buds innervated by the vagus nerve (palatal organ; figure 1). Facially innervated taste buds tended to show stronger labelling of elongate cells within the bud, but similar reactivity of elongate cells could be seen in intraoral taste buds of goldfish when using slightly shorter fixation conditions (30 instead of 60 min).

Taste buds in teleosts are organized into two distinct compartments: apically situated elongate taste cells, occupying the upper two-thirds of the taste bud, and a basal nerve plexus surrounding serotonergic Merkel-like basal cells (figure 1*e–g*; [35]). Heavy ecto-ATPase activity is evident within the basal nerve plexus above and below the Merkel-like basal cell (figure 1*e–g*). Distinct ecto-ATPase staining was evident in both compartments of the taste epithelium: elongate taste cells, within the basal neural plexus, and along the shaft of the nerve bundle below the base of the taste buds. In P2X3.2 : gfp zebrafish, gfp label is driven by the promotor for the puringergic receptor P2X3.2. In these fish, gfp-labelled nerve fibres entered the base of the taste bud to form a plexus just above the level of the serotonergic basal cell (figure 1*g*), suggesting purinergic neurotransmission in this region.

**Figure 2.**
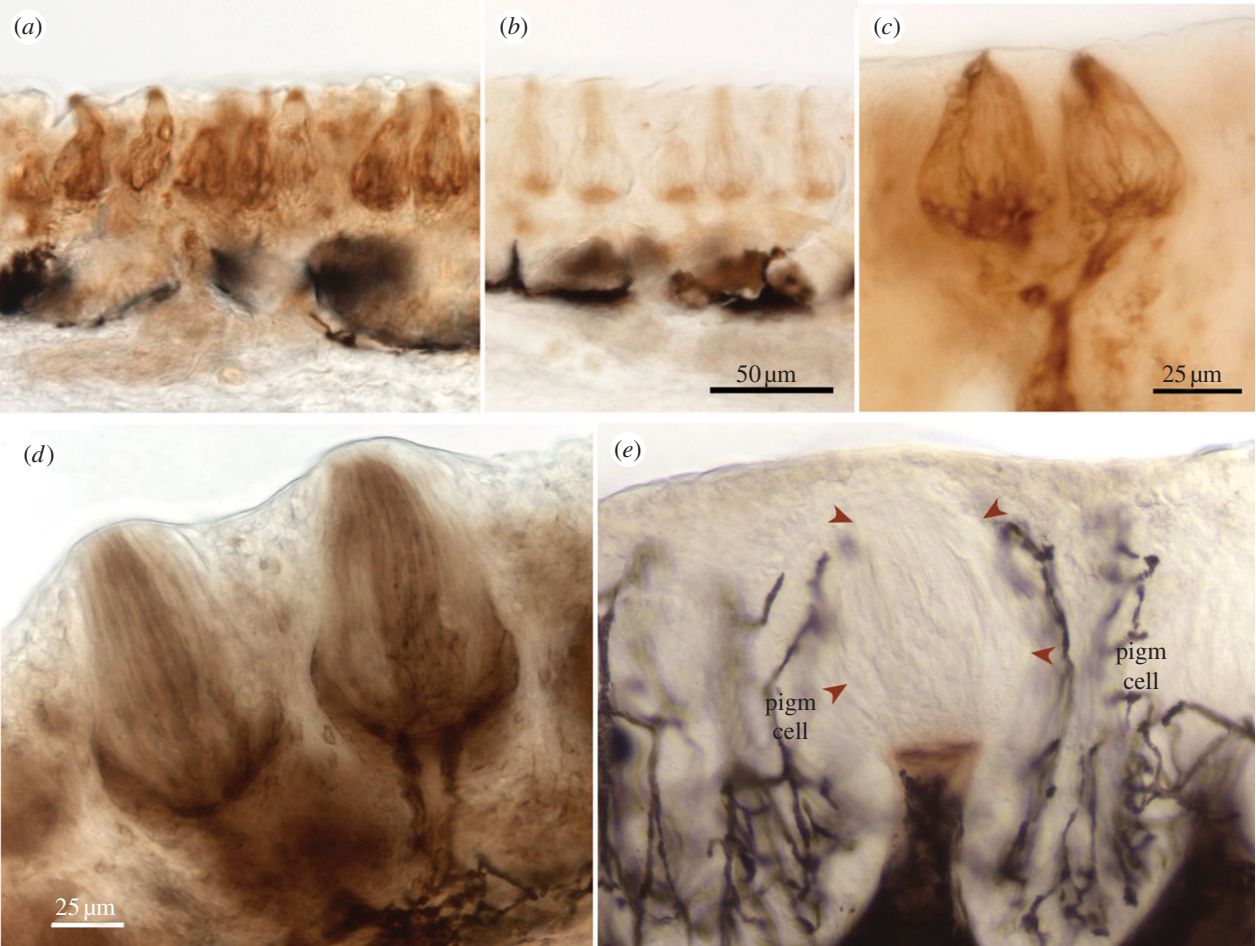
Sections through the barbel taste buds of catfishes stained for (*a*,*c*,*d*) ecto-ATPase or (*b,e*) non-specific nucleotidase. (*a–c*) *Plotosus japonicus* and (*d,e*) *I. punctatus*. Elongate cells of the taste bud are stained along with basal areas and the incoming nerve bundles. With ADP substrate (*b,e*) little or no staining is evident, other than as a basal pedestal just underneath the taste bud proper. (*e*) Arrowheads indicate the perimeter of a taste bud. Branched, black profiles (pigm cell) are melanocyte pigment cells.

In more heavily reacted or less fixed specimens of different species, numerous elongate taste cells display reaction product along their membranes. This is particularly evident in sections from the barbels of the two species of catfish (figure 2). Beneath the taste buds, heavy reaction product also is apparent along the incoming nerve bundles (figures 1 and 2). When ADP is substituted for ATP as the substrate, some reaction product remains, but this is confined to the area below the Merkel-like basal cell and includes the incoming nerve bundles. The presence of reaction product with ADP substrate indicates that at least some of this reactivity is attributable to a less specific ectonucleotidase.

**Figure 3.**
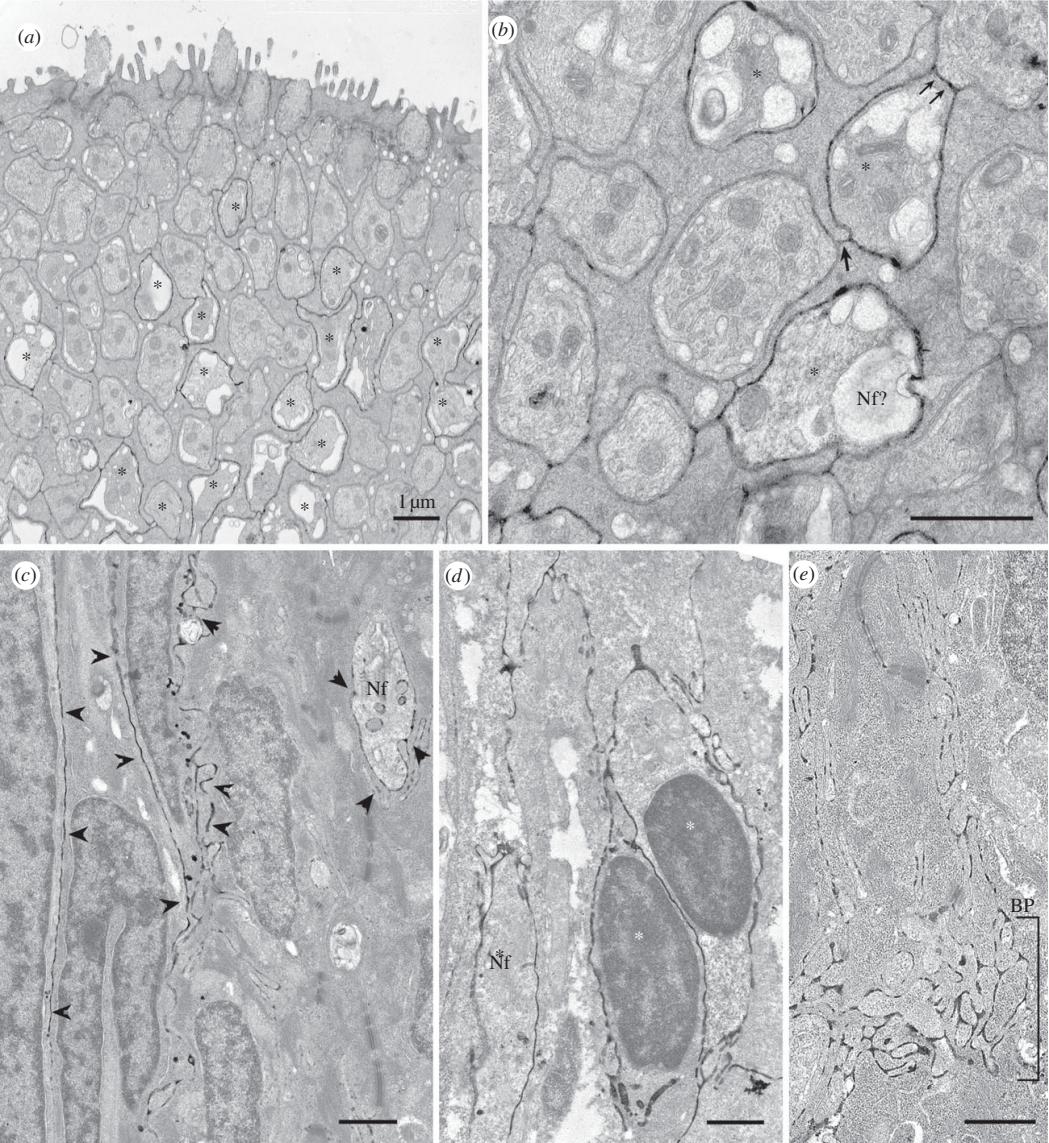
Electron micrographs of ecto-ATPase staining of taste buds from (*a,b*) the catfish, *I. punctatus* and (*c–e*) goldfish. (*a,b*) Cross sections through the apical region of a barbel taste bud showing that many but not all light cells are surrounded by reaction product. (*a*) Low magnification image showing numerous light cells (asterisks) surrounded by label at a depth of about 5 µm from the taste pore but largely devoid of label higher in the taste bud. As described by Reutter (reviewed in [27]), the light cells terminate apically in a large single microvillous process. Dark cells surround the light cells and end apically with a small tuft of microvilli. (*b*) Higher magnification view of another section from the same specimen showing details of the distribution of the reaction product. Several light cells (asterisks) are surrounded by reaction product. In one case (double arrow), a reactive light cell (asterisk) directly contacts an unlabelled light cell and the ecto-ATPase reaction product continues across this line of contact, strongly suggesting that the plasma membrane of the labelled light cell houses the ecto-ATPase enzyme. Conversely, at the junctional face of two non-reactive dark cells (single arrow), we see slight evidence of reaction product probably due to drift of the ecto-ATPase reaction product from the adjacent labelled light cells. This shows the limitations of ultrastructural analysis with this histochemical technique. (*c–e*) Longitudinal sections through the taste buds of a goldfish. (*c*) Section through the taste bud from the lip showing elongate cells surrounded by reaction product (arrowheads). In addition, a profile of a nerve fibre (Nf) is similarly surrounded by reaction product. (*d*) Palatal taste bud also revealing elongate cells (asterisks) surrounded by reaction product. (*e*) Section through the basal region of a labial taste bud showing strong reaction product surrounding many nerve fibres within the BP. The nerve fibres at this level are not surrounded by any glial elements and so the reaction product is most probably produced by ectoenzymes on the membranes of the nerve fibres themselves. This is different than the situation in rodent taste buds where the ectoenzyme is mostly associated with the membranes of the type I taste cells [13].

Ecto-ATPase staining of elongate taste cells was most apparent in tissue from lips or barbels, especially when fixation time was limited to 30 min. The apparent membrane association of reaction product seen at the light microscopic level was confirmed by electron microscopy (figure 3). Because the reaction product forms on the external face of the membrane, it is impossible to determine unequivocally at the electron microscopic level whether the enzyme activity is present on one or both of the facing cell membranes. Based on light microscopy, some elongate cells appear more reactive than others because the taste bud is not completely filled with reaction product (figure 2*a*). Similarly, in the electron microscope, some light cells are surrounded by reaction product (figure 3*a,b*), whereas dark cells and other light cells are not. The reaction product is more evident in the basal half of the taste bud, fading out as one proceeds towards the apical pore (figure 3*a*). In both goldfish and catfish, some elongate cells within each bud are surrounded by reaction product, probably indicative that these are the cells producing the ectoenzyme (figure 3*a–d*). In addition, nerve processes within the taste bud (figure 3*c*) as well as in the basal plexus (BP in figure 3*e*) are surrounded by reaction product.

In *Plotosus*, staining of the nerve bundles was especially prominent. In many taste buds, two fascicles of nerve fibres approached the bottom of the taste bud (figure 1*d*). Darker strands are visible within these fascicles, as if some elements are more reactive than others. It is not, however, apparent whether it is the nerve fibres or the ensheathing cells that display this ecto-ATPase activity.

**Figure 4.**
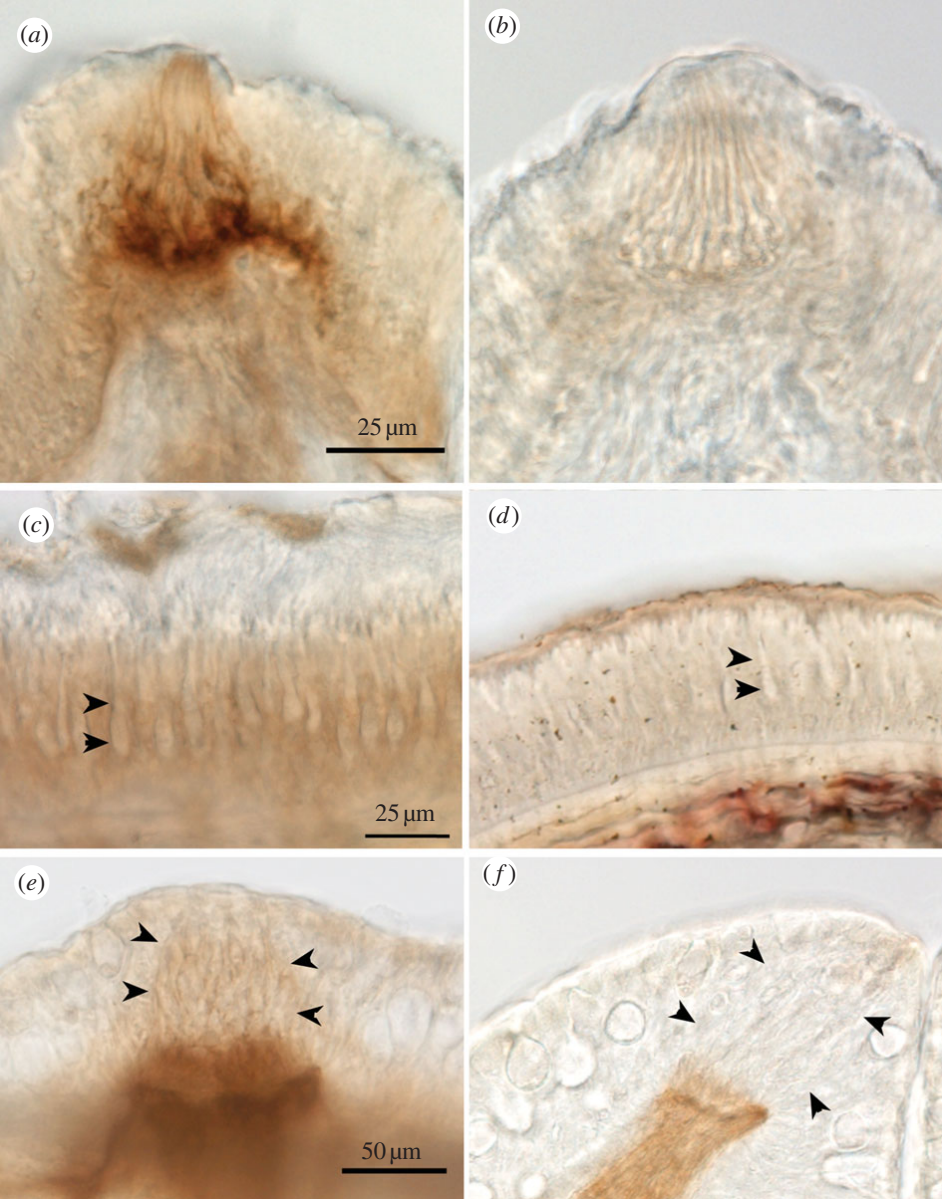
Ecto-ATPase staining (*a,c,e*) and non-specific nucleotidase staining (*b*,*d*,*f*) in tissues from the teleost searobin, *C. spinosus* (*a–d*), and from the cat shark, *S. torazame* (*e,f*). (*a*) Taste buds in the searobin stain typically as in other teleosts, showing strong ecto-ATPase staining of the basal third of the taste bud as well as of some elongate cells within upper portions of the taste bud. (*b*) The ADP substrate shows little non-specific reaction product. (*c,d*) Sections through the fin ray show numerous, unstained elongate solitary chemosensory cells (e.g. indicated by arrow heads) in both reaction conditions. (*e,f*) Taste buds (indicated by arrowheads) from the cat shark show ecto-ATPase reactivity (*e*) in the basal quarter similar to staining of the taste buds from the various teleosts. (*f*) Little non-specific nucleotidase activity is evident within the taste bud, although staining is present in the basal pedestal similar to the situation in teleosts (cf. figure 2*b*).

In the searobin, oral and buccal taste buds were similar in size to those in the barbel of catfishes and lip of goldfish, but more widely spaced. Numerous elongated cells show ecto-ATPase activity (figure 4*a*) but nothing of ADPase activity (figure 4*b*). Similar to other teleosts, substantial nucleotidase activity was present in the basal nerve plexus but some of this product was likely due to non-specific nucleotidase activity. ADPase activity could be detected in nerve bundles and strands below the taste buds, but was fainter than those in the other teleosts.

### 4.2. ATPase in taste buds of sharks

In the cat shark, taste buds are present on the lip as well as in oral, buccal, branchial and pharyngeal epithelia. Ecto-ATPase activity was present in the basal region of the buds with fainter labelling of the overlying elongate cells (figure 4*e*). Reaction product in the elongate cells was weaker than that in the Teleostei studied. ADPase activity occurred only in the nerve components (figure 4*f*).

### 4.3. ATPase in taste buds of lamprey

**Figure 5.**
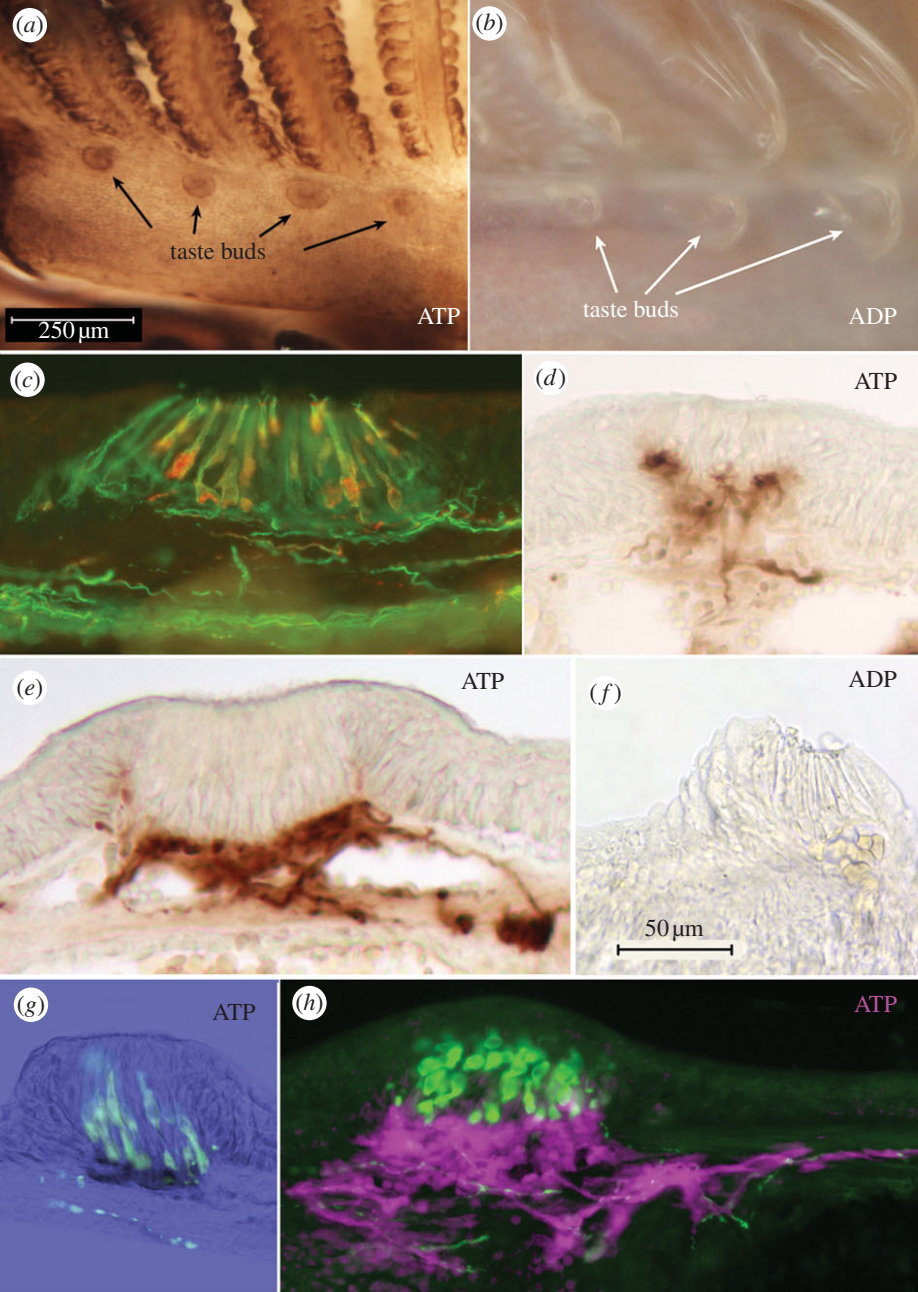
Taste buds in lamprey show specific ecto-ATPase reactivity. (*a,b*) Whole mount staining of the branchial apparatus of the lamprey showing (*a*) Ecto-ATPase staining, and (*b*) paucity of non-specific nucleotidase staining. (*c*) Staining of the taste bud for acetylated tubulin (green) and serotonin (red) shows that all cells of the taste bud are elongate cells; no basal Merkel-like cells exist (also see [37]). (*d*,*e*,*f*) Sections reveal that the specific ecto-ATPase activity (*d,e*) occurs along the basal portions of the taste bud with some lateral staining extending slightly upwards at the margins of the taste bud. With the ADP substrate (*f*) little staining is seen. (*g*) Section through a taste bud showing ecto-ATPase staining (black) and serotonin immunoreactivity (green). The basal end of the serotonin-positive cells inserts into the area occupied by ecto-ATPase staining. (*h*) An oblique section through a taste bud showing serotonin staining (green) and pseudocoloured ecto-ATPase activity (magenta). The serotonergic cells extend a basal process apparently touching and embedded within the ecto-ATPase positive regions. Fibrillar ecto-ATPase staining is present under the basal lamina in association with nerve fibres. Some serotonergic fibres also are evident, but are not associated with the ecto-ATPase staining.

Taste buds in lampreys lie along the branchial arches [36,37]. The organization of taste buds in lampreys is somewhat different than in teleosts and elasmobranchs in that lamprey taste buds lack serotonergic Merkel-like basal cells but do have elongate serotonergic cells similar to those in taste buds of mammals (figure 5*c,e*).

Reactions of whole mounts of the lamprey oropharynx showed ecto-ATPase activity in taste buds which appear as circular patches 50–100 µm in diameter along the branchial arches (figure 5*a*). Reactions of the whole mounts using ADP as a substrate showed little reactivity, even when examined in sections (figure 5*b,f*).

Sections through the taste buds showed reactivity within the basal nerve plexus (figure 5*d,e,g,h*), which in lampreys lies below the basal processes of the serotonergic cells (figure 5*g,h*). The heavy ecto-ATPase activity then lies in the region where the elongate serotonergic cells contact, and presumably synapse with, the afferent nerve fibres.

### 4.4. Hagfish Schreiner organs lack ecto-ATPase activity

**Figure 6.**
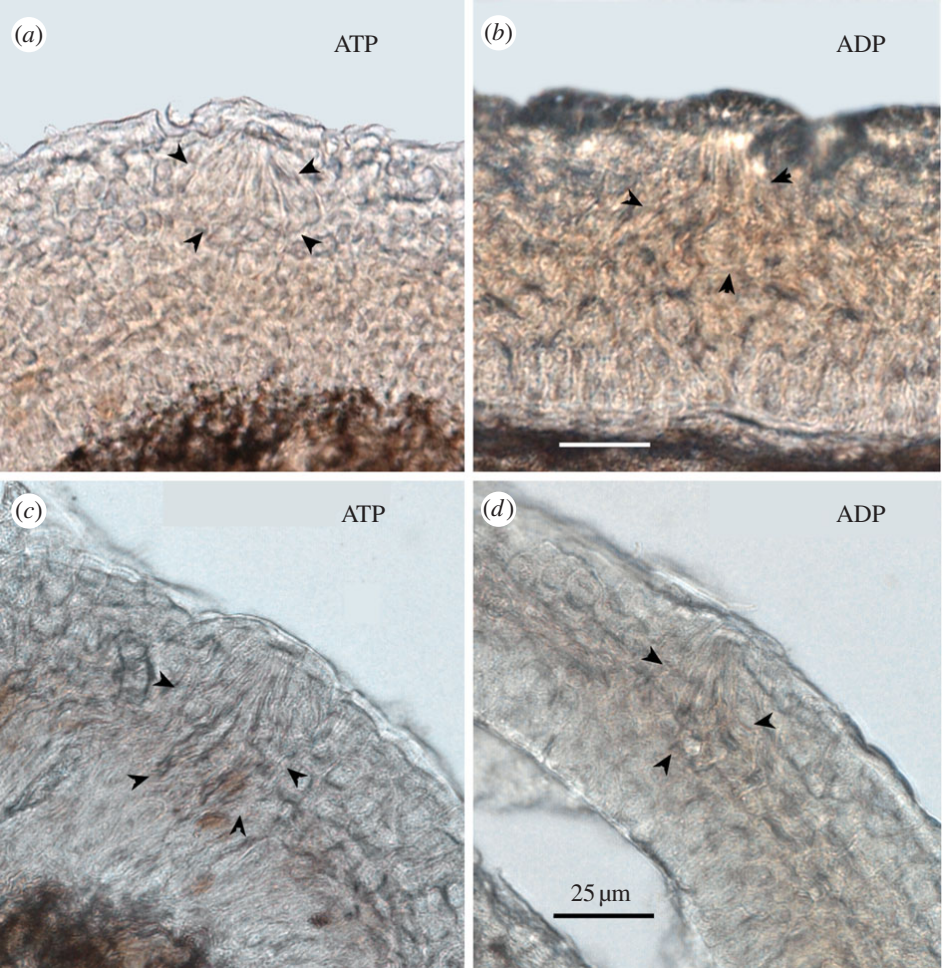
Schreiner organs in hagfish (*a,b* from tentacles; *c,d* from oral cavity) exhibit no nucleotidase activity, with either (*a,c*) ATP or (*b,d*) ADP as substrate. Arrowheads indicate the perimeter of the Schreiner organs as determined from the brightfield images. Unlike taste buds, Schreiner organs lie in the upper half of the epithelium with no obvious basal processes extending inward to reach the basal membrane of the epithelium.

Schreiner organs are widely dispersed throughout the epidermis of hagfish [4], with especially high density on the nasal and oral barbels. Neither Schreiner organs nor nerve fibre bundles nearby showed substantial ATPase or ADPase activity (figure 6). Some scattered cells deep in the epithelium showed reaction product with ATP as substrate but not with ADP. The exact nature of these cells is unclear, but they were not associated with Schreiner organs or any other obvious epithelial endorgan.

### 4.5. Solitary chemosensory cells lack ecto-ATPase activity

Solitary chemosensory cells are scattered across virtually the entire external epithelium of most teleosts [3], including on the barbels of catfishes, where SCCs are scattered between the taste buds [5]. In our preparations of catfish barbels, as shown in figure 1, ecto-ATPase activity is not apparent in the epithelium outside of the taste buds and therefore is not associated with SCCs in these locations.

The pectoral fin of sea robins has a unique specialization in which SCCs are closely packed together along the anterior three fin rays, which lack fin webbing and which thus form specialized non-taste chemoreceptor organs [38,39]. These modified free fin rays possess numerous SCCs in the epidermis, but no taste buds. Despite the high density of SCCs, the fin rays showed no detectable ATPase or ADPase activities (figure 4*c,d*). Similarly, the nerve fibre bundles that innervate SCCs showed no specific reaction product.

## 5. Discussion

In all species examined, including lamprey, an elasmobranch and all teleosts, taste buds exhibit pronounced ecto-ATPase activity. This is consistent with previous findings showing ecto-ATPase in mammalian taste buds [13,31,40,41], in an amphibian [42] and in another teleost [26]. By contrast, neither Schreiner organs in hagfish nor areas containing densely packed SCCs exhibited this trait in any species examined. Thus, the presence of ecto-ATPase appears coincident with the appearance of taste buds in the vertebrate lineage.

Mammalian taste buds are known to use ATP as a key transmitter between taste cells and nerve fibres [9], which express two ionotropic purinergic receptors, P2X2 and P2X3 [43]. The presence of ecto-ATPase is probably necessary to inactivate the ATP neurotransmitter once it is released into the extracellular space because P2X receptors will desensitize rapidly if exposed to high levels of extracellular ATP [44]. The presence of ecto-ATPase in association with taste buds of non-mammalian species suggests that all vertebrate taste buds similarly use purinergic signalling to transmit information from taste cells to nerve fibres. Indeed, zebrafish, like rodents, express P2X2 receptors on the nerve fibres innervating taste buds (figure 1*g*) [30].

SCCs are single sensory epithelial cells, present in all vertebrates from hagfish to mammals [4,8,45,46]. Like taste cells, SCCs are chemosensory endorgans consisting of secondary sensory cells, i.e. they lack an axon. Despite the similarity in function and neural relationships between SCCs and taste buds, no ecto-ATPase activity occurs in association with SCCs even in epithelia with densely packed SCCs such as the fin rays of searobins (figure 3*c*). SCCs often occur in epithelium near taste buds, as well as in respiratory passageways [4,47,48]. Yet, despite proximity to taste buds showing ecto-ATPase reactivity, the SCCs and nerve fibres innervating them lack such reactivity.

Like taste cells, SCCs have synaptic connections onto afferent nerve fibres. Whereas SCCs are innervated by either spinal or cranial nerves appropriate to the epithelium in which they reside, taste buds are only innervated by facial, glossopharyngeal or vagus nerves. Thus, SCCs are innervated by ganglion cells arising from neural crest (e.g. dorsal root ganglia), while taste buds are innervated by ganglion cells derived from epibranchial placodes [2].

Schreiner organs are sensory organs, scattered throughout the epidermis of hagfish. Schreiner organs are superficially similar in appearance to taste buds, being multicelluar aggregates composed of several types of elongate epithelial cells [4,49]. Schreiner organs are similar to taste buds as being specialized, multicellular epithelial chemosensory endorgans, but are not homologous to taste buds. They can be distinguished by several morphologic features. First, Schreiner organs lack dermal papilla, i.e. they do not sit adjacent to the basement membrane of the epithelium as do all taste buds. Secondly, Schreiner organs are innervated by spinal or cranial nerves. On the basis of these differences, Braun [4] suggested Schreiner organs are not the forerunners of taste buds. Rather, they may be a specialization of accumulated SCCs. Why hagfish lack taste buds and the associated purinergic signalling system is enigmatic. Recent molecular data indicate that hagfish are monophyletic with lampreys [50–52]. If so, the absence of taste buds probably indicates that this is not a primitive trait, but merely another of the collection of vertebrate traits that hagfish have lost during evolutionary time [53].

More than a decade ago, Finger [8] had suggested that taste buds may have evolved as an aggregation of Merkel-like sensory cells becoming associated with SCCs. This hypothesis was based in part on the similarity of morphology of the sensory cells of these two systems. But a stronger similarity was seen in the commonality of receptor mechanisms between a subset of taste bud cells and SCCs in catfish; both SCCs and a subset of taste cells express similar lectin binding indicative of an arginine receptor [5]. In mammals, the SCCs of the airways use the bitter taste (T2R) receptor cascade to detect toxins where the SCCs release acetylcholine as a neurotransmitter [47,48]. As many chemoreceptor cells in diverse organ systems rely on taste transduction cascades [48,54–59], we no longer believe that the common expression of taste receptors by SCCs and taste buds necessarily suggests a phylogenetic linkage. Furthermore, in fish, SCCs do not appear to rely on the G-protein-coupled receptor cascade characteristic of taste buds [60]. Thus, the evolutionary relationship of SCCs and taste buds is unclear.

The gustatory nerve fibres in teleosts form a plexus in the basal part of the bud surrounding the Merkel-like basal cells [15,35]. In the teleosts studied, the region within the taste buds containing the nerve fibres exhibits heavy ecto-ATPase activity. In mammalian taste buds, it is the glial-like type I cells that express the ecto-ATPase, NTPDase2. In the teleost taste buds examined at ultrastructural levels in the present study, we see specific ecto-ATPase activity associated with the plasma membranes of both elongate taste cells and nerve fibres (figure 3). Thus, in teleosts, unlike in mammals, the nerve fibres themselves appear to express a specific ecto-ATPase enzyme. In both mammals and the fishes studied herein, non-specific ectonuleotidase is associated with the nerve bundles, including glia cells, below the taste buds.

In mammals as in all species examined in the present study, the ectoenzyme associated with taste buds is highly specific for extracellular 5′-triphosphates, i.e. strongly preferring ATP over ADP [13]. The high substrate specificity is unique to this isoenzyme and accounts for the high levels of staining seen with the ATP substrate over the ADP substrate [61]. The presence of a highly specific ecto-ATPase associated with taste buds in all classes of vertebrates suggests that purinergic transmission may be one of the defining features of the gustatory periphery. The necessity for purinergic transmission is unique for the taste system although other neural systems use ATP as a co-transmitter or cofactor which modulates the effectiveness of a co-released primary neurotransmitter [62]. The use of a purinergic signal may relate to the epithelial origins of the taste system, i.e. release of ATP is a common response of epithelial cells to external stimuli [63–65]. Furthermore, in taste buds, release of ATP from type II cells is via an unusual non-vesicular mechanism involving gated hemichannels [11,12,66], with gating largely dependent on action potential-mediated depolarization of the taste cells [10]. By contrast, typical neural systems use a variety of other neurotransmitters, including acetylcholine, amino acids and amines, all of which are released via snare-protein-mediated vesicular mechanisms. In mammalian taste buds, type III taste cells make obvious morphologically typical synapses onto nerve endings [67] and use a vesicular mechanism [68] to release serotonin and GABA which act on receptors expressed by the type II cells [66,69]. Thus, taste buds appear to use both neuronal-type (vesicular) and epithelial-type (hemichannels) mechanisms to release neurotransmitter.

In other epithelial secondary receptor cells such as hair cells of the ear and lateral line organs, the sensory cells are more neuronal in terms of mechanism of transmitter release. They use vesicular release of glutamate as the primary means of transmission from sensory cell to nerve fibre [70,71]. Unlike taste buds, hair cells in these systems originate from neurogenic placodes [72], hence a vesicular, neuronal type of transmitter release is not surprising. By contrast, the more epithelial-like release of ATP via hemichannels is consistent with the origin of taste bud receptor cells from local epithelium, rather than from neurogenic placodes or neural crest [73,74].

In summary, we find that ecto-ATPase activity, indicative of purinergic signalling, is common to taste buds in all vertebrates examined to date, including lampreys, elasmobranchs, teleosts and amniotes. The commonality of taste buds and purinergic signalling mechanisms throughout the vertebrate lineage places the origin of taste buds alongside of the evolutionary origin of the earliest vertebrates. The lack of evidence for purinergic transmission from SCCs does not support the previously hypothesized relationship between taste buds and SCCs [8]. Rather, SCCs may be more primitive than taste buds, being related to the secondary sensory cells described in the epidermis of amphioxus [75,76].

## 6. Acknowledgements

The authors thank Dr Mark Voigt of St Louis University for providing the P2X3.2 : gfp zebrafish and Kristin Artinger of University of Colorado Anschutz Medical campus for helping to maintain these animals. We also express sincere thanks to Dr Takayuki Shoji of Tokai University for his help in collecting shark and hagfish. The authors thank Drs Linda Barlow and Kristin Artinger, University of Colorado Anschutz Medical Campus, for critical reading and comments on earlier versions of this manuscript. This work was supported by grants from the following sources: National Institutes of Health (RO1 DC007495 and RO1 DC009820 to T.E.F., and P30 DC04657 to D. Restrepo); and by grant-in-aids (no. 22580205) to S.K. from the Ministry of Education, Science, Sports, and Culture of Japan.
